# Stroke care in indigenous populations: A World Stroke Organization (WSO) scientific statement

**DOI:** 10.1177/17474930251347394

**Published:** 2025-05-26

**Authors:** Anna Ranta, Margaret Hart, Angela Dos Santos, Anna H. Balabanski, Susanna Ragnhild Andersdatter Siri, Carol Zavaleta-Cortijo, Seira Duncan, Amy YX Yu, CU Thresia, Tereki Stewart, Allison Kelliher, Donald Warne, Bernadette Jones

**Affiliations:** 1 Department of Medicine, University of Otago, Wellington, New Zealand; 2 Department of Neurology, Wellington Hospital, Wellington, New Zealand; 3 Department of Occupational Therapy, University of Manitoba, Winnipeg, MB, Canada; 4 Department of Medicine and Neurology, Melbourne Brain Centre at Royal Melbourne Hospital, Parkville, Melbourne, VIC, Australia; 5 University of New South Wales, South Western Sydney Clinical School, Liverpool, NSW, Australia; 6 Department of Neuroscience, Melbourne, Alfred Brain, Alfred Health, Monash University, Melbourne, VIC, Australia; 7 Center for Sami Health Research, Department of Community Medicine, Faculty of Health Sciences, UiT The Arctic University of Norway, Tromsø, Norway; 8 Intercultural Citizenship and Indigenous Health Unit (UCISI), School of Public Health and Administration, Cayetano Heredia Peruvian University, Lima, Peru; 9 Social and Human Working Group, International Arctic Science Committee, Akureyri, Iceland; 10 Department of Medicine (Neurology), University of Toronto, Sunnybrook Health Sciences Centre, Toronto, ON, Canada; 11 Institute for Mental Health and Neuro Sciences, Kozhikode, India; 12 Faculty of Education, Health, and Psychological Sciences, Victoria University of Wellington, Wellington, New Zealand; 13 Johns Hopkins University, School of Nursing and Bloomberg School of Public Health, Baltimore, MD, USA

**Keywords:** Stroke care, stroke outcomes, Indigenous, co-design, traditional, community, first nations, health equity

## Abstract

**Background:**

Indigenous Peoples have been reported to experience higher rates of stroke, poorer access to high-quality acute and rehabilitation stroke services, and worse post-stroke outcomes compared to dominant cultures residing in the same countries. The aim of this statement is to summarize available evidence on access barriers contributing to these inequities, effective solutions that have been deployed and tested, and present key recommendations to advance the field.

**Methods:**

We conducted a scoping review searching Medline, Embase, CINHAL, PubMed, Scopus, and Informit Indigenous Collection using the broad search terms “stroke” and “Indigenous” without date restriction until 1 August 2024. We screened 673 unique titles, 96 abstracts, and 80 full-text papers of which we retained 41. We added 10 additional key references known to authors. Articles were analyzed to identify key cross-cutting themes.

**Results:**

We identified five key themes: (1) Historical context, colonization and racism; (2) wholistic strength-based approaches to health, well-being, and recovery; (3) communication, health literacy, and cultural safety; (4) Indigenous knowledge systems, research principles, and community-led action; (5) achieving local acceptance versus wide generalizability.

**Recommendations:**

Key priority areas, detailed in the form of 11 specific recommendations and based on six core values, include improving stroke service responsiveness, Indigenous Peoples empowerment, and Indigenous research support to better meet the needs of Indigenous Populations globally. The statement has been reviewed and approved by the WSO Executive Committee.

## Graphical abstract

**Graphical abstract:**
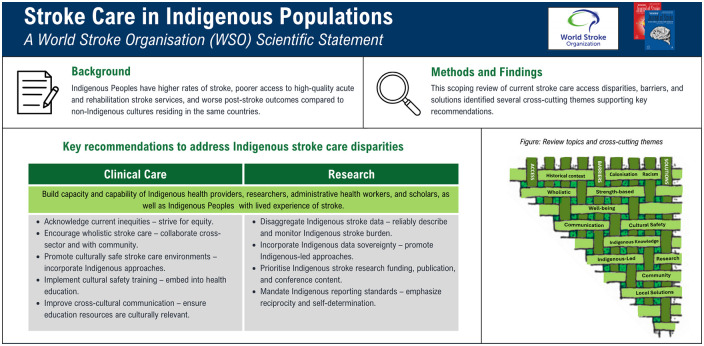

## Purpose

The purpose of this scientific statement is to provide guidance to the scientific and clinical stroke community on how to approach the care for individuals with stroke who self-identify as Indigenous or belong to Indigenous communities, to support strategies to eliminate current inequities both at individual and system levels, and guide research approaches.

## Background

Stroke is one of the leading causes of death and adult disability globally.^1^ Indigenous Peoples have been reported to experience increased stroke risk factors and disparities in stroke incidence, with stroke often occurring at a younger age.^2–5^ A few Indigenous populations have been reported to experience overall excellent health-status and similar or even lower stroke incidence compared with the dominant culture; however, these benefits appear linked to their ability to retain traditional practices and life-styles, benefits that are increasingly at risk of being lost and replaced by accumulated stress related to loss of land, culture, and inability to maintain traditional practices.^6–8^ Regardless of incidence and risk factors, Indigenous Peoples experience reduced access to high-quality acute and rehabilitation stroke care^9–16^ and poorer post-stroke outcomes including higher mortality.^2,4,11,12,17,18^

## Methodological complexities

Conducting a “scientific” review on the topic of stroke care in Indigenous Populations is not straight forward due to incongruence around definitions, data collection, and methodologies.

### Defining “Indigenous”

The word Indigenous has origins in Latin *indigena*, meaning “native” or “sprung from the land” and the World Health Organization defines “Indigenous Peoples [as being] custodians and practitioners of unique cultures and ways of relating to people and the environment. They possess social, cultural, economic, and political characteristics that are distinct from those of the dominant societies in which they live.”^19^ As a grouping, “Indigenous People” have been recognized by the United Nations. The United Nations Declaration on the Rights of Indigenous Peoples (UNDRIP) was passed as a non-binding resolution by the UN in 2007 and consists of 24 pre-ambular provisions and 46 articles that set out the minimum standards for the survival, dignity, and well-being of Indigenous Peoples throughout the world, including rights related to governance, health, community, culture, language, lands, territories and resources, and education (see Table 1 for key highlights).

**Table 1.** A summary of a sample of articles from the UN Declaration on the Rights of Indigenous Peoples (UNDRIP) with direct relevance to healthcare.

Articles 3, 4, and 5	Emphasize the right to self-determination in political, social and cultural affairs, including health.
Article 18	Recognizes the right of Indigenous Peoples to participate in decision-making in situations that will affect them and to allow Indigenous Peoples to maintain their decision-making methods.
Article 21	Sets out the right of Indigenous Peoples to improvement of their economic and social conditions including education, housing and health.
Article22	Pays particular attention to the rights of special needs of those most vulnerable in the community, including Indigenous elderly, women, children and disabled.
Article 23	Sets out the right of Indigenous Peoples to development, including their right to be actively involved and administer programs that affect them.
Article 24	Recognizes the right of Indigenous Peoples to traditional medicine and the maintenance of their traditional practices including conservation of relevant resources. This right also includes the right to access health services for Indigenous Peoples.

However, it should be noted that, the term “Indigenous” is problematic as it suggests a single cohesive group of Indigenous populations yet the estimated 476 million Indigenous Peoples living across more than 90 countries worldwide are incredibly diverse even within a single country.^20^ In addition, not all Peoples who meet the WHO definition and UNDRIP inclusion refer to themselves by that term. Other “umbrella” terminology is used such as “First Nations,” “Natives,” “Adavasi,” and “Aboriginal” to name just a few. To complicate things further, many Indigenous Peoples refer to themselves by names in their own language not consistently matching the names given to them by others. Which terms are used, and which are preferred, may also change over time with negative connotations associated with some more than others. From a global perspective, “Indigenous” has emerged as the currently preferred single umbrella term, when referring to these communities collectively and some important commonalities do exist. For the purposes of this article, we considered publications that were identified using the search terms “Indigenous” and “stroke” (see below for more detail on search strategy) excluding only those where “Indigenous” clearly referred to the dominant culture as in “indigenous Africans” in comparison to “African Americans.”

### Impact of discrimination on data validity

In addition to the definition challenge, accurate population and disease estimates are complicated by the fact that many groups meeting the above criteria prefer not to use the term “Indigenous” due to factors including historical discrimination and marginalization.^21^ This is the case, for example, for many Ryukyuans (primarily in present-day Okinawa, Japan). Although they are recognized as Indigenous by the United Nations and other organizations such as the International Work Group for Indigenous Affairs and the Minority Rights Group, most do not self-identify as Indigenous, with implications for health research.^22^ Similarly, governments may not recognize the existence of Indigenous populations.^23^ Many do not collect self-identification of Indigenous status in census or in public records as this is regarded as sensitive information.^24^ Others continue to prioritize assimilation.^23^ However, most Indigenous groups increasingly advocate for their recognition as Indigenous and demand ethnic disaggregated health data capture to help identify and address ongoing health and social disparities.^23,24^ There is also increasing emphasis on, and advocacy of, Indigenous data-sovereignty relating to increased involvement regarding how data about Indigenous people are designed, collected, overseen, stored, and used.^25^

### Differences in research methodologies

Finally, given cultural differences, “scientific research” in the Indigenous space can look very different than “dominant” or non-Indigenous research, and some initiatives will never result in journal publication due to parallel Indigenous knowledge systems often preferring other forms of dissemination such as verbal presentations to local communites.^26,27^ While the dominant mainstream scientific method focuses on the health of individuals and operates from a predominantly biomedical model of health, many Indigenous populations operate from a collectivist model, focussing on a wholistic community-specific approach that supports not only individual physical health, but also the mental and spiritual well-being that contribute to and also rely on the overall well-being of the extended family and community.^28^ This Indigenous well-being model is less suited to being assessed by Western positivist methodologies such as randomized controlled trials or even large scale observational studies. As a result, the available literature on “Indigenous stroke” is generally dominated by non-Indigenous researchers using dominant scientific approaches “doing research *on* Indigenous Peoples.” This carries a significant risk of disempowerment and perpetuating historical injustices. Alternatively, the literature may take the form of more Indigenous congruent ethnographic, narrative and/or qualitative methodologies that are preferably community driven and focus on “*by* and *for* Indigenous Peoples” that may be dismissed by the dominant culture as “inferior” or even entirely “unscientific.” This tension is profound and should be a primary focus for reconciliation.

## Approach and writing panel

With these considerations in mind, we aimed to interweave various knowledge systems to offer a collective way forward, acknowledging the goodwill of members of the dominant culture to support the health of Indigenous Peoples in their pursuit of well-being. However, it is also important that non-Indigenous audiences approach this topic with cultural humility and a genuine willingness to engage with Indigenous perspectives.

To support our effort, we have formed an international panel of researchers with expertise in the fields of stroke care, implementation science, health equity, and Indigenous health. Our panel included senior stroke clinicians, stroke researchers, and individuals with extensive experience in collaborating with Indigenous communities to improve Indigenous stroke care delivery and conduct stroke research. Importantly, more than 50% of the panel self-identifies as Indigenous and belonging to Indigenous communities with extensive research experience in the Indigenous health space. We deliberately prioritized Indigenous researchers with expertise in Indigenous stroke and wider relevant health research as we saw the Indigenous voice and expertise as most critical for this work. Prioritizing the involvement of Indigenous voices provides essential perspectives and represents an important step toward decolonizing current Indigenous research efforts. Many of our Indigenous researchers also hold important governance roles within their community, one is a stroke survivor, and another an Indigenous Elder. These diverse perspectives add further depth to the expertise of the panel and strengthen the community voice.

To ensure our work meets Indigenous cultural publication standards, we adhered to the “Consolidated criteria for strengthening reporting of health research involving Indigenous Peoples” (CONSIDER) framework (see Supplement).^29^

## Scope of evidence reviewed

Most of the available literature focusses on the epidemiology of stroke in Indigenous populations describing incidence and risk factors from a dominant scientific paradigm. Instead of reiterating recently published reviews on this topic^2,17,30^ and restating the problem, this statement explores underlying causes, identified solutions, and proposes a way forward. Due to the nature of this topic and the associated methodological challenges, the panel agreed that a systematic review employing standard evidence grading was not the appropriate methodology. Instead, we conducted a systematic literature search to underpin a narrative report.

Given the challenges around defining “Indigenous,” the panel discussed appropriate search terms. The initial proposal was to include various potential synonyms for the term “Indigenous” such as “First Nations,” ‘Aboriginal, “Natives,” and so on. However, it became clear that this would prioritize Western colonized populations that preferentially use these terms over groups that prefer using region specific or locally unique terms such as the South Asian “Adavsi,” and Japanese “Ainu.” When we considered adding these search terms, we realized many more would need to be added to achieve completeness. We considered adopting a recently compiled exhaustive list of Indigenous Peoples in high-income countries.^17^ However, this would have missed Indigenous Peoples in low- and middle-income countries, some of which were not even included under UNDRIP, and realized that “completeness” was not, in fact, achievable. In addition, capturing all historical research involving Indigenous People would have required including terminology that was never approved by Indigenous People and is now viewed as inappropriately disempowering or even racist, and the panel preferred not to perpetuate the use of such historic terms. Reassuringly, trialing a few broader searchers, beyond the term “Indigenous,” for example, including “Adavasi,” or “First Nations,” did not, in fact, substantially change the number of identified relevant papers.

Therefore, after much deliberation, the panel agreed to limit the systematic search to the terms “Stroke” and “Indigenous” as the globally most accepted collective term for these diverse populations at this time in history, and supplement with relevant papers from other sources based on the panel’s expert opinion. We feel that this achieved the best balance between two conflicting scientific and cultural paradigms, and optimally achieves our goal to “interweave” approaches while also achieving reasonable data saturation. To further optimize the search, we expanded it to five databases including the Informit Indigenous Collection.

On 1 August 2024, we searched Medline, Embase, CINHAL, PubMed, Scopus, and Informit Indigenous Collection using the broad search terms “stroke” and “Indigenous.” We screened 673 unique titles, 96 abstracts, and 80 full-text papers of which we retained 41. We focussed on primary research although retained some key review articles to inform the discussion. We included papers of all types of methodology and highlighted publications that demonstrated cultural consideration in line with the CONSIDER framework as part of their methodology. We added additional key references known to authors (n = 10).

We collated articles into tabular format grouping to allow the reader easy reference by *article topic*. However, to interweave non-Indigenous and Indigenous methodologies, we present the narrative aspect of this report according to *cross-cutting themes* that we identified throughout the reviewed papers, regardless of topic type, aiming for a more wholistic thematic presentation. Within each cross-cutting theme we highlight specific access barriers, Indigenous principles and values, and present case studies of implemented solutions. We conclude with a set of recommendations.

## Results of the literature search

Of the 41 full-text articles and policy papers included in this report, the majority came from Australia (n = 17), followed by Aotearoa New Zealand (n = 10), and much fewer from USA (n = 4), Japan (n = 4), Canada (n = 3), and South Asia (n = 3). We found single articles from Brazil, South Africa, Taiwan, Korea, Norway, and Southeast Asia, and included three multi-national reviews. Most of the evidence and resultant recommendations are based on experiences from high-income, English-speaking countries with a colonial past. However, there is clear evidence from the broader literature that there are also very similar challenges faced by Indigenous Peoples in other parts of the world including many parts of Asia, South America, and Africa and we have included some of these examples where feasible.^15,23,24,31–33^

**Figure 1.**
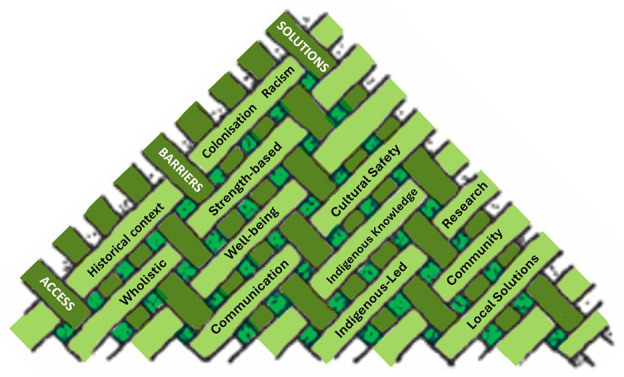
Interweaving approach to presenting literature review results. Dark green bands = article groups as displayed in Tables 1–3; light green bands = cross-cutting themes used to structure the narrative aspect of the paper starting immediately below.

Tables 2–5 summarize publications grouped by stroke care “Access,” “Barriers,” and “Solutions.” However, the below narrative summary is structured according to five cross-cutting themes that emerged from most if not all articles regardless of table category. These themes are “Historical context, colonisation and racism,” “Wholistic strength-based approaches to health, well-being, and recovery,” “Communication, health literacy, and cultural safety,” “Indigenous knowledge systems, research principles, and community-led action,” and “Achieving local acceptance versus striving for wide generalisability.” The reader should not attempt to marry up the table headings to cross-cutting themes. Data are presented deliberately in two formats to interweave dominant scientific and Indigenous approaches, with an emphasis on an Indigenous wholistic approach presented in the narrative portion of this statement (Figure 1). Table 5 summarizes key review articles and policy papers.

**Table 2.** Articles on stroke care **access.**

Author, year	Study type/setting	Relevant findings
Kilkenny et al., 2012^13^	Cohort study, Australia	Indigenous Australian patients with stroke received a reduced quality of care in hospitals and experienced worse outcomes than non-Indigenous patients.
Tiedeman et al., 2018^14^	Cohort study, Australia	Investigations and post-discharge care of indigenous ischemic stroke patients is inferior to non-indigenous patients.
Dos Santos et al., 2020^34^	Cohort study, Australia	Indigenous Australian patients with stroke experience delayed presentation attributed to under recognition of stroke symptoms when admissions to stroke units care were compared with non-Indigenous patients.
Cochrane et al., 2022^35^	Cross-sectional, Australia	Indigenous Australian patients with stroke were assessed to be more commonly using informal rather than formal standard communication rehabilitation tools presumably because standard tools are not culturally appropriate.
Thompson et al., 2022^12^	Cohort study, Aotearoa, New Zealand	Non-Europeans, especially, Indigenous Māori, had poorer access to key stroke interventions and experienced poorer post-stroke outcomes.
Denison et al., 2023^11^	Cohort study, Aotearoa New Zealand	Found ethnic disparities in care and outcomes following stroke, independent of traditional risk factors including socioeconomic disadvantage. Findings most prominent among Māori.
Samuels et al., 2020^36^	Cohort study, Aotearoa New Zealand	There was equal access to acute stroke reperfusion therapies and those receiving treatment achieved similar outcomes comparing different ethnic groups, including Indigenous Māori, in Northern New Zealand.
Fujiwara et al., 2018^9^	Cross-sectional, Japan	Found regional disparities in access to stroke hospitals in Hokkaido, the home of Indigenous Ainu population of Japan.
Souto et al., 2021^15^	Cross-sectional, Brazil	Self-declared black, Asian, indigenous and other individuals have worse access to post-stroke rehabilitation when compared to self-declared white people.
Yu et al., 2021^16^	Cross-sectional, USA	Disparities in access to stroke centers were greater in nonurban areas than urban areas with greater census representation of elderly, American Indian, uninsured, and low-medium income populations.

**Table 3.** Articles describing barriers and exploring principles and values when caring for Indigenous People.

Author, year	Study type/setting	Study population	Relevant findings
Kelly et al., 2022^37^	Qualitative, interviews/focus groups, Australia	N = 6 patients N = 78 rehab providers	Aboriginal people report making positive lifestyle changes but report significant unmet rehabilitation needs highlighting the need to advocate for improved communication and more flexible rehabilitation delivery.
Armstrong et al., 2019^38^	Qualitative, interviews Australia	N = 32 patients n = 18 family/carers	Need for improved communication focussing on cultural safety, identity, language, and social/health context, involving family and care closer to ancestral country to promote recovery.
Cochrane et al., 2023^39^	Qualitative interviews, Australia*	N = 7 Indigenous Health Liaison Officers	Identified need for strong connections with both family and country and health professionals to promote recovery as well as consideration of spirit and emotions to support a culturally safe care environment for people with acquired communication disorders.
Quigley et al., 2019^40^	Qual/quant surveys, Australia	N = 24 patients N = 10 carers N = 70 providers/NGO	Identified the need for inclusive, coordinated and culturally responsive approach to Aboriginal and Torres Strait Islander stroke care that values the client, their family, and community. Developing the Aboriginal and Torres Strait Islander health workforce was emphasized, as well as the need for follow-up closer to home. Importance of appropriate communication and culturally secure care was stressed.
Eustace et al., 2023^41^	Kaupapa Māori research, qualitative interviews, Aotearoa New Zealand*	N = 4 Patients N = 2 Family N = 5 Speech pathologists	Six themes: (1) tautoko (support); (2) Kaupapa Māori (Māori approach); whanaungatanga (relationships); tino rangatiratanga (autonomy); (5) taiao (environment), and (6) kōnekeneke (change). They concluded the need to prioritize strong collaborative relationships, offer more autonomy to Māori communities, support Māori SLT** development, increase public awareness of challenges, and encourage change in the wider healthcare system. They drew parallels to Australian studies.
Hersh et al., 2015^42^	Quantitative/Qualitative survey, Australia	N = 112 Speech and language pathologists	Reported insufficient knowledge about Indigenous culture, lack of support and lower level of confidence working with Indigenous clients. A desire for more flexible services, good access to interpreters and culturally appropriate assessments and treatments delivered in a culturally appropriate setting.
Legg and Penn, 2013^43^	Ethnographic/interviews, South Africa	N = 15 nurses	Emphasized the importance of cultural background and social context on the experience of aphasia and an awareness of cultural differences can aid in promoting recovery. Concepts beyond the biomedical aspects such as spirituality and perceived causality in the specific cultural context can shed light on how people understand their condition, make meaning of language loss, the strategies that people employ in order to seek help, and the responses of families and communities to people living with communicative disability
Liao et al., 2023^44^	Qualitative interviews, 3 locations, Taiwan	N = 24 dyads	A sense of place was found to contribute to identity and use of own language and strong kinship ties with care provided within Indigenous people’s own native community facilitated post-stroke recovery.

* Indicates publications that reported cultural consideration in line with the CONSIDER framework as part of their methodology; **SLT = speech and language therapist.

**Table 4.** Articles exploring solutions, including specific interventions, to optimize Indigenous Stroke Care.

Author, year	Study type/setting	Intervention	Relevant findings
Peake et al., 2021^45^	Participatory action research (PAR), topic yarning framework,* Australia	Culturally appropriate health research resources	The PAR using the Research Topic Yarning (RTY) framework developed culturally relevant health resources. This approach ensured the community’s voice was central, fostering confidence and empowerment in driving the process in collaboration with healthcare professionals. The project emphasized community control and partnership with mainstream health services, illustrating the importance of family and community in health interventions.
Armstrong et al., 2017^46^	Qualitative focus groups and interviews (with health providers), Australia	To develop an acquired communication screening tool, n = 4 FG, n = 4 interviews	Following a literature review, clinicians with experience working with aboriginal people were consulted. Findings stressed the need to include family views, use a conversational ‘yarning’ based approach, reduce direct questions, use culturally appropriate images including familiar yet not stigmatizing imagery (i.e. not kangaroo), acknowledge time constraints.
Armstrong et al., 2019^47^	Validation pilot, Australia	Acquired communication referral screening tool, n = 38	Tool looks promising in ability to offer culturally secure screening for Aboriginal people to identify need for specialist referral. Challenges included participant recruitment, scheduling appointments across vast distances, availability of Aboriginal research assistants.
Armstrong and colleagues, 2024^48–52^	Stepped-wedged randomized controlled trial, Australia	Aboriginal-led model of care stroke and traumatic brain-injury n = 312 (per protocol)	The Healing Right Way project, inspired by recommendations from “Missing Voices,” introduced the role of Aboriginal Brain Injury Coordinator to support Aboriginal adults with brain injury and their families for 6 months post-injury. ABICs liaise with health services, advocate, provide support, and educate about brain injury and available services. In addition, cultural security workshops aimed at improving interactions within rehabilitation services were conducted for clinical staff. The final results have not been published although were presented at the ANZSO conference in 2023 and were reported as equivocal on the primary and most secondary outcomes although showed improvements in achievement of minimum processes of care (MPC) for the intervention group of patients. Surveys with patients at 26 weeks post-injury found that those in the intervention group were more satisfied with hospital services than those in the control group. Multiple associated publications have appeared to date including a process evaluation referenced here.
Armstrong et al., 2023^52^	Summary of multiple studies (mostly qualitative), Australia	Use of Aboriginal yarning to facilitate speech recovery.	The Wangi (Talking) and Yarning Together projects, employed a speech-language pathologist and an Aboriginal co-worker to work with Aboriginal individuals with acquired communication disorders in a yarning framework within their home environment. Yarning, a dialogic process rooted in deep cultural significance, facilitates reciprocal and mutual interaction, promoting a clinical yarning framework where non-Aboriginal clinicians engage Aboriginal clients in a culturally relevant manner.
Brewer et al., 2020^53^	Qualitative longitudinal pilot, Aotearoa New Zealand	Online course for speech and language therapists caring for Māori; n = 11	Impact on participants included a better understanding of inequities and racism, the need to self-reflect and change practice, sharing learnings with others, and improving relationships and communication with Māori clients (and families), the need for learning Māori language and practices, and engaging more with Māori colleagues. A need to ensure continued application by sharing with others and taking responsibility.
Møller et al., 2023^54^	Culturally informed community PAR; surveys and interviews, Canada^* †^	Community rehabilitation worker program; n = 51 survey; n = 47 focus group; mix of elders/workers/learners	Themes informing the curriculum: importance of culture, tradition, spirituality, and religion, mastery of native language, community re-engagement and re-integration post event, mental health support specific for First Nations Elders, local/regional transportation assistance, options for Elders to gather in the community.
Morii et al., 2019^55^	Economic modeling study, Japan	Develop a drive and retrieve model of care	Development of a drive and retrieval system deemed likely to enhance regional equity and cost effectiveness of ischaemic stroke treatment in Hokkaido within existing resource.
Park et al., 2014^56^	Descriptive, Korea	Integrating traditional healing into acute and post-acute stroke care	Report positive experiences in their service following implementation of a model where in the acute stage, neurologists or neurosurgeons take charge, with Korean medicine doctors as secondary physicians taking a greater lead sub-acutely offering acupuncture, moxibustion, and herbal prescriptions.
Hill et at, 2017^57^	A multiyear community case study in Ontario, Canada	Engaged First Nations children to develop culturally attuned resources for stroke education.	Addressed stroke education needs among First Nations children aged 11–13 years. Guided by the Northwestern Ontario Regional Stroke Network Aboriginal Advisory Committee, the study developed age-appropriate and culturally relevant educational products, ensuring methods reflected the cultural values of participating communities.
Harwood et al., 2012^58^	Randomized controlled trial, Aotearoa New Zealand	Inspirational Māori video stories plus a ‘Take Charge Session’ n = 172	While the videos were not associated with improved outcome, the single, ‘Take Charge After Stroke intervention,’ was found to result in measurable meaningful post-stroke improvements. It was subsequently shown to also be effective in a non-Māori/Pacific population. Take Charge is a talking therapy that puts the patient at the center of identifying meaningful recovery goals and was initially co-designed incorporating Māori and Pacific patient stories and tested in Māori and Pacific communities in New Zealand.
Harwood et al., 2012^59^	Randomized controlled trial. Aotearoa New Zealand	Hua Oranga outcome instrument developed for Māori N = 172	The Hua Oranga instrument, developed for Māori people with mental illness, uses a wholistic view of Māori health to determine improvements in physical, mental, spiritual and family domains of health and showed good responsiveness and adequate psychometric properties in Māori and Pacific people after stroke. Its simplicity, relative brevity, minimal cost and adequate psychometric properties should favor its use in future studies with both Māori and Pacific people. Suggestions are made for refinements to the measure. These should be tested in a new population before Hua Oranga is recommended for general use in a clinical setting.
Ranta et al.,^60^	Descriptive, Aotearoa New Zealand	Predominantly Indigenous consumer panel to advise on national level hyper-acute stroke service	The New Zealand National Stroke Clot Retrieval Improvement Project engaged a more than 50% Māori consumer panel that helped co-design several patient resources including consent leaflets, a patient video outlining the stroke journey, and contributed to service specifications around telestroke services to reduce persisting access inequities. This effort included an overnight stay on a marae (traditional meeting house) for the entire panel (including non-Māori members) to meet on ‘safe land’ to help empower Māori voices.
Oser et al., 2013^61^	Collaborative development and evaluation with before and after telephone surveys, USA	Culturally specific public awareness campaigns for signs and symptoms of stroke	The Montana Cardiovascular Health Program, in collaboration with tribal health departments, developed and disseminated awareness materials via local media and demonstrated the effectiveness of culturally tailored health communication.
Murad et al., 2011^62 †^	Regular fields surveys, Pakistan^†^	Cataloging of indigenous knowledge medical plants	The majority of the local inhabitants in the study area depend on plants to treat human ailments and this study aimed to record the indigenous knowledge and medicinal uses of plants before the ethnomedicinal information are lost. The folk medicinal uses of 75 plant species were recorded for various human ailments. The juice of the flower, Lythraceae, is given twice a day in the dose of 8–10 ml for treatment of stroke.
Jagtap et al., 2006^63^	Regular field surveys, India	Cataloging of indigenous knowledge medical plants	Researchers identified 66 medicinal species. Nirguda (*Alectra parasitica*), the plant is crushed and boiled in water which is used for bathing, and a cup of fresh plant juice is taken to cure stroke; Tarota (*Cassia tora*), dry fruit powder taken with a cup of milk once a day for 3 days to cure stroke; Pingvel (*Celastrus paniculatus*), pickle of flowers is eaten twice a day to cure stroke; half a cup of leaf decoction is taken for 10 days to cure menstrual disorders; Mu (*Madhuca longifolia*) Flower, powder mixed with oil of Sesamum orientale, and 50 g paste is taken daily for 1–2 months to cure stroke; Turshi (*Chlorophytum borivilianum*), dry tuber powder is mixed in ghee prepared from cow’s milk along with coconut pulp and 20 g of the mixture is taken daily to cure stroke; Tulas (*Ocimum tenuiflorum*), two spoons of fresh leaf juice is taken twice a day to get relief from stroke; Choso (*Semecarpus anacardium*), pickle of flowers is eaten with food to cure stroke; *Thespesia pop*ulnea, a cup of bark juice is taken once a day to cure stroke.
Walter et al., 2021^64^	Descriptive, Rural Australia	Air-mobile stroke unit to reduce geographic barriers	This work is in progress. The paper describes the development of an air mobile stroke unit service model to reduce geographic barriers disproportionately affecting Aboriginal Australians.
Wakuta et al., 2020^65^	Descriptive, Rural Japan	Drip and ship network to reduce access barriers for acute stroke	This descriptive piece highlights the achievable gains of helicopter transfers between remote islands in Japan where especially Indigenous Ryukyuans are disadvantaged due to geographic distances. Similar models have been explored in other geographical dispersed areas across the globe and the intervention is not Indigenous specific.

* Indicates publications that demonstrated cultural consideration in line with the CONSIDER framework as part of their methodology.

† Indicates publications covering broader grounds than stroke.

**Table 5.** Key Review Articles, Statements, Policies, and Guidelines.

Author, year	Type of Publication	Relevant findings
New Zealand Stroke Foundation, 2010^66^	Chapter in National Stroke Guidelines on Stroke in Māori, Aotearoa New Zealand	Makes several recommendations: Māori participation in decision-making, planning, development, and delivery of stroke services; quality audits to identify disparities in stroke care by ethnicity; develop Māori specific outcomes, Māori specific post-stroke education using stories, culturally appropriate services including development of Māori workforce and culturally safe non-Māori workforce that considered cultural beliefs, practices, and preferences; allowing person with stroke to define ‘whānau (family)’, goal setting to include family, emotional, physical, cultural, and social needs; research programs employing Kaupapa Māori research methodologies that provide evidence on access, quality, and outcomes for Māori with stroke and develop and evaluate Māori specific stroke interventions.
Heart and Stroke Foundation of Canada, 2024^67^	Heart and Stroke Foundation Strategy, Canada	Principles: Understanding that meaningful partnerships and collaborations must be based on mutual respect; Recognizing unique circumstances and geographic challenges of Indigenous people in Canada; Understanding that activities must be community driven and controlled; Recognizing that the social and economic determinants of health are at the core; Adhering to the Truth and Reconciliation Commission Report by addressing health-related calls to action and reconciliation; Ensuring that “nothing about us – without us” is the fundamental ethic of our ambitions.
Penn et al., 2018^68^	International Roundtable N = 9 stroke SLT researchers), Australia, South Africa, Aotearoa New Zealand, Canada	Core values to underpin decolonization of Speech and Language Practice: (1) Trust and relationship building; Trust and relationship building; (2) Two-way dialogue between service providers and communities; Reflective practice by healthcare practitioners in regard to their assumptions and practices; (4) Acknowledgment and recognition of (ongoing inequity); (5) Incorporation of cultural understandings relating to family, community, and local cultural protocols into routine clinical practice; (6) Recognition that indigenous peoples are best placed to work with their own communities, hence the imperative to work closely with Indigenous colleagues and grow Indigenous speech-language pathology workforce.
Balabanski et al., 2024^17^	Systematic review on Incidence in Indigenous Populations, Very High Human Development Index Countries*	Indigenous populations experience generally, although not universally, higher stroke incidence, highlighting concerns about data accuracy and lack of CONSIDER guideline use. Some evidence of slower rate of incidence decline, stroke at younger age, male predominance. Proposed multi-factorial reasons including social and economic disadvantage resulting in poorer health outcomes due to poor living conditions, education access, health care access, and changes in food environment, exacerbated by stroke occurring at a younger age, cultural and language barriers. Recommending capacity and capability building of Indigenous workforce and developing culturally safe non-Indigenous workforce and services guided by the Indigenous community.
Dos Santos et al., 2021^2^	Narrative review of stroke in Indigenous Peoples, Worldwide*	Found higher incidence, higher mortality, presentation at a younger age, more risk factors, and lower treatment rates. Analogous to the general health of all populations, the cause of stroke and its outcomes are the result of complex interactions between factors that act at societal, service, and individual levels. Colonization, including the cultural and material losses associated with it, is a major indirect driver of disparities in lifestyle, biological and other risk factors for stroke in Indigenous Peoples. In addition, structural racism and the inability to access culturally safe education, employment and health care, further contribute to stroke health inequities for Indigenous Peoples globally.
Harris et al., 2015^4^	Systematic review of stroke in American Indians and Alaskan natives, USA	Prevalence of stroke is more common, risk factors are higher, presentation occurs at younger age, and mortality data in AI^**^ and AN^**^ are significantly compromised due to misclassification and low study power. When accounting for these methodological issues mortality is the highest among ethnic groups in United States. Identified barriers include delayed care, rurality, access barriers highlighting historical issues around colonization related trauma including geographic displacement and forced assimilation resulting in disconnection to from land, culture, and language, highlighting the notion of a delayed ‘transition’ to modern lifestyle to explain ‘rise’ in CVD is not evidenced based. Recommended improved data accuracy, large-scale studies with purposeful oversampling, needs assessments, and development of more effective, culturally congruent interventions.
Ranta et al., 2023^69^	Narrative Review of stroke in Māori, New Zealand*	Provides summary of historical context, current disparities in incidence, age, service access, and outcomes, and highlights policy solutions at government level that emphasize Indigenous rights, values, priorities, and approaches to healthcare, presenting solutions developed in partnership with Māori researchers and community aiming to transition all such research to being entirely Māori-led.
Blacker et al., 2019^70^	Editorial on Indigenous stroke care calling for change, Australia	Summarizes barriers including issues related to communication breakdown, non-aligned models of health, and institutional racism. Recommending cultural security training for health staff, involvement of Aboriginal coordinators, improved communication, support materials, and interpreter access, improved post-discharge support that includes sufficient flexibility to meet Aboriginal community needs, taking into account need for proximity to ancestral land and inclusion of wider family.
Sharma et al., 2023^5^	AHA Scientific Statement on maternal cardiovascular health in AI/AN* individuals, USA ^†^	Reviews epidemiology AI/AN** pregnant women highlighting dipropionate disease burden, gaps in data, recommending integrated care delivery models, integrate community voice/traditional knowledge/cultural safety into care organization, increase funding support, improving health education, and consider values, philosophy, and historical trauma to build trust and respect, improve communication, and tailor care delivery.
Peake et al., 2019^71^	Systematic review of n = 22 studies on effectiveness of health education, Australia ^†^	Identified the need for collaborative relationships, community ownership, cultural sensitivity and a lack of evaluation of current resources. Barriers to achieving appropriate resources included distance, time, funding, and the need for cultural competency in mainstream health.
Minority Rights Group, 2018^31^	Report on Indigenous Ainu, Japan ^†^	Summarizes the impact of colonization, including mistreatment, introduction of diseases, land loss, forced assimilation, loss of culture and language (which was forbidden), and economic and social marginalization affecting the Indigenous Ainu population in Japan.
Norwegian National Human Rights Institution, 2020^24^	Policy statement, Norway ^†^	This Norwegian report is concerned with Indigenous Sami health data, outlines the human rights-based approach to indigenous statistics, explaining why disaggregated data are essential for the implementation of indigenous rights, but also how to safely manage indigenous peoples’ data and prevent its misuse.
Thresia et al., 2022^32^	Review of Indigenous health, South Asia ^†^	This article is not stroke specific but highlights that despite South Asia’s promising social inclusion processes, ongoing social and health inequalities leave Indigenous populations excluded and marginalized with associated poor health care access, quality of care, and outcomes. Unequal power relations, poor policy responses, loss of land and culture, and other factors are discussed as adversely impacting Adivasi populations. Impacts of colonization and the need to look beyond biomedical factors are emphasized.
Asia Indigenous Peoples’ Pact et al., 2019^23^	Briefing Paper, ASEAN Countries, Southeast Asia ^†^	Summarizes challenges faced by Indigenous People in ASEAN countries who report often not being recognized by their governments and desiring the right to self-determination; rights to land, territories, and resources; rights for free, prior, and informed consent; rights to development; cultural rights.
Pedersen et al., 2010^33^	Narrative essay, Peru ^†^	This article examines some of the long-term health outcomes of extreme adversities and the ways in which social inequalities and idioms of distress are historically and socially produced in the Peruvian context.
United Nations^72^	United Nations Declaration on the Rights of Indigenous Peoples ^†^	The Declaration on the Rights of Indigenous Peoples (UNDRIP or DOTROIP) is a legally non-binding resolution passed by the United Nations in 2007 and updated in 2013 and 2018. It delineates and defines the individual and collective rights of indigenous peoples, including their ownership rights to cultural and ceremonial expression, identity, language, employment, health, education, and other issues.

* Indicates publications that demonstrated cultural consideration in line with the CONSIDER framework as part of their methodology; ^†^ Indicates publications covering broader grounds than stroke. They have been included where either stroke specific evidence is lacking or where general needs and rights of Indigenous People’s, also highly relevant to those with stroke, are discussed. ^**^ AI = American Indian; AN = Alaska Native.

## Key cross-cutting themes

### Historical context, colonization, and racism

Our own history influences our approaches to health. Western stroke clinicians and scientists typically focus on biomedical aspects of health to explain stroke risk and outcomes. However, there is little evidence to indicate that Indigenous Peoples worldwide share specific biological attributes that explain the common patterns of increased stroke risk factors,^2–5^ stroke incidence, stroke care access barriers,^9–16^ and poor post-stroke outcomes.^2,4,11,12,17,18^ Some of the shared disparities may be attributed to geographic access challenges, as many Indigenous Peoples reside rurally. We found evidence of effectively addressing, or planning to address, rural access to stroke care, especially related to reperfusion access disparities, through well-organized hyper-acute stroke networks and novel technologies such as air mobile stroke units.^9,36,57^ Addressing geographic access barriers to acute and rehabilitation services is important and should continue.

However, geographic distance cannot fully explain Indigenous stroke disparities. Many studies have effectively controlled for geographic and other biological patient factors, concluding that significant aspects of access inequity could only be explained by historical and resultant social factors.^11,12,70^ Nearly all of the reviewed literature from across the globe identified that many, if not all, of the stroke-related health inequities experienced by Indigenous Peoples today relate to socioeconomic, cultural, and environmental consequences attributed to colonization, marginalization, systemic racism, and underlying power hierachies.^2,4,5,69^ Some of the consequences relate to socioeconomic disadvantages including poverty and lack of educational opportunity. Others relate to service provision that does not align with cultural values (see below). Finally, there is persistent distrust due to inter-generational trauma related to forced assimilation and destruction of culture and language, forced migration and loss of ancestral land and territories, systemic mistreatment and subjugation, widespread violations of their human rights, and other forms of systemic racism.^2,4,69,70,72^

The parallels around systemically enforced assimilation by separating Indigenous children from their families and placing them in often very unsafe boarding schools is remarkable across nations and cultures, as are governmental policies of assimilation, forbidding the use of Indigenous languages, cultural customs, knowledge systems, and healing practices resulting in a dramatic power imbalances and the loss of self-determination.^2,4,23,32,53,69,70,73^

One example of persistent modern systemic racism is the ban, in some countries, on collecting disaggregated ethnicity data that precludes the identification of the stroke burden and access disparities. Even where these data are collected, common misclassification of ethnicity has led to profound misrepresentation of stroke burden that can present a highly inaccurate picture downplaying disparities.^4,24,74^

Similarly, the mistrust of healthcare and research settings has been perpetuated by untrustworthy and unethical research that has exposed Indigenous populations to undue harms.^75^ It is our collective responsibility to rebuild eroded trust.^76^

### Wholistic^1^ strength-based approaches to health, well-being, and recovery

An important aspect for Indigenous populations is the value placed on extended family and community,^38-40,44^ connection to ancestral land,^38,39,69,70^ and a general preference for a wholistic approach to well-being that considers not only physical, but also spiritual, emotional, mental and community health and well-being.^5,39,41,43,54,59^ Prioritizing these needs in the setting of a sterile inpatient ward away from their own community, with little space to accommodate large families and limited therapies that focus exclusively on physical health create obvious barriers.

Many Indigenous Peoples view health as the presence of well-being rather than the absence of disease, creating incongruence with concepts such as “rehabilitation” which generally focusses on current deficits instead of future aspirations. While goal setting is a well-supported approach to achieving outcomes in dominant health systems, the type of priorities goals set may differ substantially between cultures yet there may be very little interest in exploring the consideration of Indigenous patient centered outcomes.^37,79^

The dominant health system thus continues to evoke paternalistic approaches with the expectation for people from different cultures to simply accept dominant approaches and it is probably not surprising that uptake and trust often remain limited; this is especially likely on the backdrop of the historical practices mentioned above. Insisting that “dominant approaches work better” risks being perceived as patronizing and insensitive and fails to support a move toward decolonization and restorative practice. On top of this, the presumption that dominant practices are necessarily superior is inaccurate, as evidenced by persisting gaps in stroke incidence and outcomes between Indigenous and non-Indigenous populations worldwide.^4,12,17,80^

We found several examples of stoke interventions tailored to Indigenous needs that achieve improved post-stroke outcomes compared to usual approaches. One randomized controlled trial tested the benefits of a co-designed “talking therapy” called “Take-Charge After Stroke” that incorporates Indigenous values and aspects of health beyond physical recovery. This intervention achieved significantly reduced disability compared to usual care.^58^

The Wangi (Talking) and Yarning Together intervention, employed a speech-language pathologist and an Aboriginal co-worker to work with Aboriginal individuals with acquired communication disorders in a “yarning framework” within their home environment. Yarning is a dialogic process rooted in deep cultural significance, that facilitates reciprocal and mutual interaction. It promotes a clinical yarning framework where non-Aboriginal clinicians engage Aboriginal clients in a culturally relevant manner.^52^

A group in Korea reported positive experiences in their service following implementation of a model where in the acute stage, neurologists or neurosurgeons take charge, with Korean medicine doctors as secondary physicians taking a greater role in the sub-acute phase offering acupuncture, moxibustion, and herbal prescriptions.^56^

There are more opportunities to integrate traditional, wholistic therapies, such as therapeutic massage, prayer, ceremonial practices and other forms of spiritual healing, and traditional native or herbal remedies, which have been actively promoted by some guidelines.^5,66^ As interest in traditional and wholistic healing approaches continue to grow, it is essential to support efforts that prevent the loss of traditional Indigenous medicinal knowledges. Such efforts are reported in South Asia where researchers are working with local communities to document traditionally used healing plants.^62,63^ However, when undertaking such projects it is critical that Indigenous communities are not exploited or harmed as part of this process. These efforts can be very extractive, result in “biopiracy,” with little to no acknowledgment, compensation, or support for participating Indigenous communities.^81^ For instance in Okinawa, southern Japan, although locals have contributed to a plethora of studies and documentaries exploring their exceptional longevity, local life expectancy has been rapidly declining over the past few decades, raising questions of what practices responsible and ethical research entails.^7^

### Communication, health literacy, and cultural safety

Many reviewed papers stressed the importance of improving communication between stroke healthcare providers and Indigenous Peoples and their families with or at risk of stroke.^5,37,38-40,42,53,61,70,77^ Many, especially elder Indigenous Peoples, are not fluent in the country’s dominant language and interpreter services can be critical to improve communication and stroke care. However, often even more subtle challenges present major barriers. Educational materials, whether paper or digital, are often very “white” and mono-cultural in their imagery including the people in the pictures and the environment and activities they display (e.g. NIHSS “cookie theft” picture https://www.ninds.nih.gov/health-information/stroke/assess-and-treat/nih-stroke-scale). This can create unexpected barriers in communication including in the evaluation of stroke symptoms and outcomes. Armstrong et al.^47^ developed a culturally secure and tailored communication assessment tool for Aboriginal Peoples with stroke and found a high degree of validity in their pilot to accurately identify the need for specialist referral. They employed more of a storytelling approach than yes/no questions. When developing such tools, it is imperative to involve Indigenous Peoples with lived experience to inform the design and ensure stigmatizing imagery (e.g. kangaroos for Aboriginal Peoples) is avoided. An exemplary approach to this was demonstrated by Peake et al.^45^ using Participatory Action Research (PAR) and the Research Topic Yarning (RTY) methodology, putting community voice at the center.

Several of the papers we identified highlighted delays in presentation to hospitals as a key reason for access disparities.^4,64,82^ This has also been identified to be of relevance to non-Indigenous people and has resulted in much attention on public awareness campaigns such as FAST (i.e. “Face,” ‘Arm, “Speech,” and “Time” to guide stroke recognition and emphasize urgent action). While these efforts are undoubtedly helpful in general and potentially for Indigenous populations, many such “health literacy” attempts have failed to consider the cultural relevance of such materials.^61,83^ In Aotearoa New Zealand, the Stroke Foundation and Ministry of Health partnered with the Health Promotions Agency in a Māori-led team that prioritized culturally relevant materials, which was associated with improved uptake of the message.^84^ The Montana Cardiovascular Health Program collaborated with tribal health departments to develop and disseminate culturally tailored awareness materials,^61^ and the Northwestern Ontario Regional Stroke Network Aboriginal Advisory Committee guided the development of an age-appropriate and culturally relevant educational program to raise stroke awareness among children aged 11–13 years.^57^

These successful efforts highlight a key shift in the approach to “health literacy,” de-emphasizing the presumed lack of “health literacy” among Indigenous Peoples when mainstream messages are slower to resonate and instead ensuring messages are tailored culturally appropriately to achieve the desired effect. In other words, we must acknowledge that much of the responsibility lies with the “communicator” if communication is ineffective, not simply the audience. This notion also plays a crucial part in the provision of “culturally safe” or “secure” stroke services. Many publications stressed the importance of provider awareness of not only cultural differences traditionally associated with the concept of gaining “cultural competence,” but to shift the focus to “cultural safety,” which requires associated self-reflective humility that acknowledges historical trauma, continued power imbalances, one’s own cultural background and associated biases, and the need to create space for Indigenous Peoples to retain autonomy and self-determination.^2,5,38,40,47,66,69^

**Figure 2.**
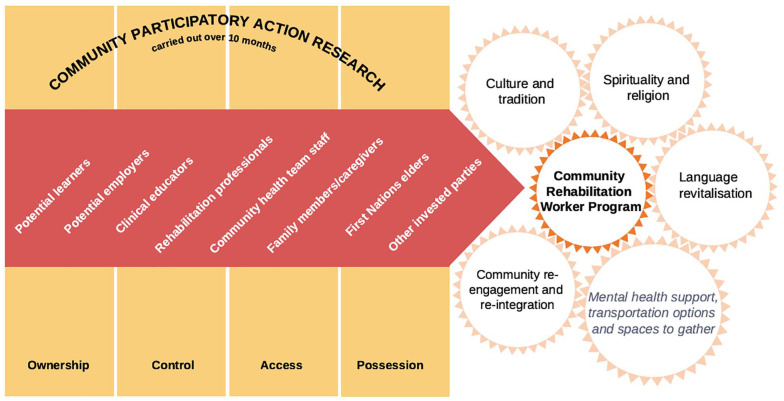
Ontario Community Stroke Rehabilitation Program. H Møller et al (2023) reformulated the Community Rehabilitation Worker program in Northwestern Ontario using the Community Action Participatory Research process involving First Nations elders, their caregivers and family members. The new training program is co-facilitated by a First Nations elder and more appropriately supports First Nations elders’ rehabilitation by incorporating components of local culture, language, spirituality and community re-integration to promote health, well-being and quality of life. Mental health support, transportation options and places to gather were also important.

To this end, cultural safety programs have emerged, and some of these have been evaluated, indicating significant utility. Brewer et al.^53^ developed an online course for speech and language therapists caring for Māori patients, and their evaluation found that participants gained a better understanding of health inequities, the need to self-reflect, change practice, and improve relationships and communication with Māori. Møller et al.^54^ (Figure 2) used participatory action involving Indigenous elders in Canada to develop a community rehabilitation worker program. Engagement with community resulted in the prioritization of culture, tradition, spirituality, religion, mastery of native language, community re-integration, and mental health support specific to First Nation’s Elders as key aspects for workers to internalize when offering rehabilitation programs. Some service improvement activities will relate to both Indigenous and non-Indigenous communities and when involving people with lived experience of stroke it can be important to ensure membership is at least half Indigenous, instead of a “token” single person who is often then drowned out, marginalized, and dominated by the non-Indigenous members perpetuating power imbalances. A successful example of such a power-balanced, lived-experience panel took place in Aotearoa New Zealand as part of the National Stroke Clot Retrieval Service Improvement Project.^60^

These efforts are more effective if they are systematized, incorporated into general and preventive health care curricula, and continued during specialist stroke training. Examples of the former exist,^29^ although we are not aware of any examples specific to stroke. It should be noted that attaining “cultural safety” may not be achievable in culturally incongruent settings, and for this reason some panelists proposed the terms “culturally safer” or “cultural humility” to be more appropriate moving forward.

### Indigenous knowledge systems, research principles, and community-led action

It is crucial to prioritize cultural safety or cultural humility training for stroke health workers as the first step in decolonizing stroke services and taking restorative action. However, true transformative change will only occur when Indigenous health and research leadership is fully entrusted to Indigenous Peoples themselves. The importance of self-determination cannot be overstated.

As already demonstrated, Indigenous communities have existent health systems that sit within broader Indigenous knowledge systems that include their own traditionally based research methodologies. These are often entirely incongruent to the dominant analytic reductionist approaches^26^ and despite tremendous advances achieved by mainstream health systems, an expectation that Indigenous Peoples completely abandon their own knowledge systems, perpetuating erasure and assimilation, is unsurprisingly viewed as paternalistic, unacceptable and inappropriate. This is especially significant as key domains, such as spirituality, connection to land, ancestry, community and wholistic well-being have been excluded from dominant models. In effect, the current imposed approach to health means well-being, as defined from an Indigenous worldview, is essentially impossible to achieve.^2,69,70^ Table 6 presents a comparison between the two approaches.

**Table 6.** Comparisons of key aspects of approaches to health and health research.

Current dominant ‘Western’ approaches	Common themes in Indigenous approaches
• Biomedical focus with emphasis on physical health	• Wholistic focus with equal emphasis on physical health, mental health, spiritual health, family/community health, connection with land and environment
• Interventions focussed on the individual	• Interventions often involving family and/or community, or emphasizing the collective
• Interventions created by the researcher and/or clinicians	• Interventions actively involve community for co-creation
• Interventions and research results aiming to be widely generalizable	• Interventions accepted as needing to be tailored to a specific community
• Quantitative methodologies prioritized especially randomized controlled trials	• Qualitative methodologies prioritized especially ‘story telling’
• Preferentially led by an expert in stroke medicine	• Preferentially led by a researcher with both cultural and clinical expertise that match the needs of the Indigenous community

While attempts to integrate the two approaches is laudable and should be encouraged, it is also important that Indigenous Peoples have the autonomy to progress their own systems of care, using their own research methodologies, that is led by Indigenous researchers themselves.^2,66,69,70^ The concept of community-led research is critical in the Indigenous space. Multiple studies have emphasized the need for an approach of “by Indigenous for Indigenous” instead of imposing systems designed by clinicians and researchers from the dominant health care and knowledge systems. This principle forms the foundation of many Indigenous research approaches and is well-described in the influential work on “Decolonising Methodologies” by Linda Tuhiwai Smith.^26^ Two examples of Indigenous research methodologies include the Aotearoa New Zealand Kaupapa Māori framework based on earlier work by Smith et al.^85^ and applied to stroke care by Eustace et al.^41^ and the Australian RTY framework^87^ as used by Peake et al.^45^ Both tools incorporate storytelling as a data collection methodology, emphasize community participation, and other core values such as trust, relationships, and reciprocity. The application of such a framework was well demonstrated in the consideration and evaluation of the Hua Oranga outcome instrument in stroke survivors to measure more wholistic and culturally appropriate outcomes that are actually meaningful to Māori.^59^ It is entirely conceivable that non-indigenous researchers would have never considered that different approaches to analyses or outcome measures may be required for culturally distinct populations.^86^

To facilitate meaningful change, the dominant scientific community must consider Indigenous research as highly valuable and meaningful, and as such support these transformative research approaches if equitable outcomes in stroke services are to be achieved.^26^ However, even if such support was universal, a separate major barrier to advancing these concepts is a persistent shortage of Indigenous scholars generally and in stroke care specifically. There is a wealth of evidence that diversifying the clinical workforce improves outcomes for minority populations,^88^ and this has also been shown to be critical among Indigenous Peoples.^89,90^ Efforts such as affirmative action are often viewed as controversial or even unfair. However, this perspective emerges primarily when the historical context is not considered, inequitable outcomes are ignored, and population health is deprioritized over individual rights. These are dominant colonial perspectives that require reflection and recognition that workforce development programs should focus on effective clinical outcomes among diverse populations.

Even once an academic degree is achieved, Indigenous clinicians and researchers have to navigate a potentially unfamiliar and non-preferred paradigm and often face ongoing challenges. This relates not only to the methodologies used, but also a de-emphasis on journal publication, the individual ownership and leadership of research being devalued in collectivist participatory programs, and local publications, favored by Indigenous scholars, being viewed of less general interest by dominant academic institutions than large multi-center and multi-country research. Even simple aspects of promotion applications such as identifying international referees can be extremely challenging if their research is highly focussed on the local community. Finally, the few Indigenous researchers we do have are frequently overburdened by requests to serve on a variety of committees, projects, and studies. This leaves them with much less time for their own research priorities.

Important first steps include removing barriers for new Indigenous researchers to allow building greater capability and capacity among the Indigenous research community and training Indigenous Peoples with lived experience to contribute to and conduct their own research. It is essential to provide strong and supportive mentorship programs that advance these individuals in a culturally safe environment.

A key step for scientific communities is to value and even prioritize Indigenous research and methodologies for stipend and grant funding allocation, journal publication, and conference presentations. At a minimum, funders and journals could ensure adequate reporting guidelines are followed when funding, reviewing, or publishing research involving Indigenous Peoples.^29^ The CONSIDER guideline is an excellent option for journals to adapt as it incorporates all of the above outlined principles.^29^

### Achieving local acceptance versus striving for wide Generalizability

One of the key challenges in Indigenous stroke care and research is the conflict between prioritizing local development and ownership on one hand and on the other, a desire for large sample sizes, across wide reaching geography, to achieve greater study power and generalizability. The former better meets the Indigenous community needs, the latter achieves higher scientific community accolades and potentially greater and more efficient scientific impact. The need for local ownership is partly driven from local differences that, perhaps, are more important in a model that focusses less on biomedical and more on social mechanisms of health and well-being.^26^ Ignoring these factors is problematic when imposing interventions on an Indigenous community. However, undoubtedly there is also a component of distrust when it comes to introducing models developed by outsiders who have historically taken rather than shared and while large scale multi-national randomized controlled trials may result in very accurate scientific data, the impact will be massively curtailed if the results are not accepted by local communities.^7,75,76^ This may, in fact, explain some of the delays in knowledge translation across other marginalized communities globally and contribute to widening disparity gaps in some areas.

Perhaps the optimal approach is to continue with large scale studies, but place more effort on partnering with Indigenous Peoples, including those with lived experience, from the start to give them a voice when it comes to designing interventions and also determining meaningful outcome measures. The dominant scientific community could also consider being more open to allowing amendments to non-Indigenous “evidence-based treatments” to meet local needs without always demanding additional costly randomized controlled trial evidence. For example, as observed during the pandemic, Indigenous-led health approaches were leveraged to support direct healthcare for community members.^91^ More importantly, clinicians and academics need to place more value on resourcing and testing novel interventions within an Indigenous research framework before forcing local adaptation or dismissing the lack of adoption of non-Indigenous medicine as attributable to limited understanding.

These are challenging concepts, but they will need to be considered and embraced if there is a genuine desire to help improve Indigenous health and achieve equity in stroke outcomes.

### Recommendations

The totality of the literature we reviewed emphasizes the need for innovation and transformative change in our approach to Indigenous Peoples at risk of and experiencing stroke by acknowledging historical and societal context as well as cultural differences and being more open toward incorporating Indigenous knowledge systems. This along with the need for locally tailored and culturally specific approaches to stroke care preclude sweeping recommendations advocating for a specific intervention or approach. Rather, there is a need for underpinning values and principles on how the stroke community ought to approach Indigenous Peoples with stroke and their supporting communities.

A recent international roundtable proposed a set of such core values for managing Indigenous Peoples with acquired communication disorders, predominantly stroke, that nicely summarizes the concepts discussed in detail above.^67^ We have adapted these values slightly to provide a solid foundation to guide broader stroke care in Indigenous Peoples:

1. Acknowledgment and recognition of ongoing inequity.
2. Reflective practice by stroke practitioners regarding assumptions built into practice.
3. Trust and relationship building.
4. Incorporate cultural understandings relating to extended family, community, and local cultural protocols into routine clinical practice.
5. Two-way dialogue between service providers and communities.
6. Recognize that health initiatives involving Indigenous communities should be led by Indigenous Peoples.

**Figure 3.**
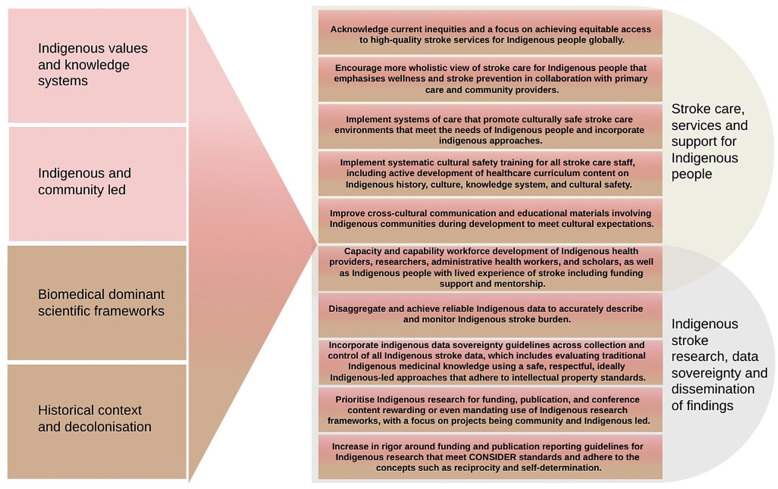
Summary of Key Recommendations.

Based on the presented literature and supported by their own experience, the panel makes the following recommendations to drive effective service improvement and research activities:

1. While recognizing the strengths and resilience of Indigenous Peoples, openly acknowledge current inequities and focus on achieving equitable access to high-quality stroke services for Indigenous Peoples globally;
2. Encourage a more wholistic model of stroke care for Indigenous Peoples that emphasizes wellness and stroke prevention in collaboration with primary care, community providers and Indigenous communities;
3. Address current knowledge gaps and develop, evaluate, and implement systems of care that promote culturally safe stroke care environments across all phases of stroke care that meet the local needs of Indigenous Peoples and incorporate Indigenous approaches;
4. Provide systematic cultural safety training for all stroke care staff, including active development of healthcare curriculum content on locally relevant Indigenous history, culture, knowledge system, and cultural safety;
5. Improve cross-cultural communication and educational materials using a co-design methodology with Indigenous communities during development to meet cultural expectations;
6. Increase workforce capacity and capability of Indigenous health providers, researchers, administrative health workers, and scholars, as well as Indigenous Peoples with lived experience of stroke including funding support and mentorship;
7. Disaggregate and achieve reliable Indigenous data to accurately describe and monitor Indigenous stroke burden;
8. Incorporate Indigenous data sovereignty guidelines across collection and control of all Indigenous stroke data, which includes evaluating traditional Indigenous medicinal knowledge using safe, respectful, ideally Indigenous-led approaches that adhere to intellectual property standards;
9. Prioritize Indigenous research for funding, publication, and conference content rewarding or even mandating use of Indigenous Research Frameworks, with a focus on projects being community and Indigenous led;
10. Increase rigor around funding and publication reporting guidelines for Indigenous research that meet CONSIDER (or similar) standards and adhere to the concepts such as reciprocity and self-determination.
11. Finally, we call for organizations to actively support collaboration between Indigenous and non-Indigenous Peoples with lived experience, clinicians, and researchers from different geographic locations across the globe, to actively explore ways to address tensions between dominant scientific and Indigenous knowledge systems including the “locally grown” versus “optimized generalizability” challenge. (Figure 3)

In addition to the above clinical and stroke research priorities, it is important to bear in mind that many social determinants of health, such as a safe living environment, education, access to clean water and affordable food, play a critical role in vascular health. Current systemic discrimination often reaches beyond the health into the social and political sector. We should, within our roles in the stroke community, acknowledge these issues as intrinsically unfair and advocate finding solutions at individual or organizational levels. Of importance, the issues discussed in this article focus on common themes identified via our literature search across Indigenous populations, but some aspects will not universally apply and there will always be important differences between these diverse cultures to consider. All broad recommendations need to be considered within the local context.

We also acknowledge that many of the above principles may also be relevant to other marginalized populations and those from different cultures who have immigrated to a new country. We encourage others to apply these principles to these broader populations as appropriate. However, it is equally important to recognize that Indigenous Peoples have a unique experience in the way that changes were forced upon them by others invading their lands and imposing their culture. Many Indigenous Peoples have been subjected to extreme forms of forced assimilation, cultural extinction or near extinction, and systematic abuse. This has resulted in extreme distrust of the dominant culture. The dominant cultures have designed and govern current health systems and to rebuild the trust that has been destroyed the outlined recommendations are especially critical.

We believe that through open, genuine, trustworthy, respectful, and culturally safe engagement, Indigenous and non-Indigenous researchers and practitioners can build strong collaborative relationships locally, regionally, nationally, and internationally to learn from each other and achieve high-quality and equitable stroke care and outcomes for all people.

## Notes

1. We deliberately chose the spelling “wholistic” to emphasize “wholism” as in referring to the “whole” rather than parts a concept that is central to Indigenous knowledge systems.

## References

[1] Thayabaranathan T Kim J Cadilhac DA , et al. Global stroke statistics 2022. Int J Stroke 2022; 17: 946–956. 35975986 10.1177/17474930221123175 PMC9980380

[2] Dos Santos A Balabanski AH Katzenellenbogen JM , et al. A narrative review of stroke incidence, risk factors and treatment in indigenous peoples of the world. Vessel Plus 2021; 5: 21.

[3] Dos Santos A Mohr K Jude M , et al. Stroke risk factors and outcomes in indigenous verse non-indigenous Australians. Heart Lung Circulat 2019; 28: S57.

[4] Harris R Nelson LA Muller C Buchwald D. Stroke in American Indians and Alaska Natives: a systematic review. Am J Public Health 2015; 105: e16–e26. 10.2105/AJPH.2015.302698 PMC4504319 26066955

[5] Sharma G Kelliher A Deen J , et al. Status of maternal cardiovascular health in American Indian and Alaska Native individuals: a scientific statement from the American Heart Association. Circ Cardiovasc Qual Outcomes 2023; 16: e000117. 10.1161/HCQ.0000000000000117 37254753

[6] Willcox DC Willcox BJ Todoriki H , et al. The Okinawan diet: health implications of a low-calorie, nutrient-dense, antioxidant-rich dietary pattern low in glycemic load. J Am Coll Nutr 2009; 28 Suppl: 500s–516s. 10.1080/07315724.2009.10718117 20234038

[7] Ryall J. Japan: What’s behind Okinawans’ falling life expectancy? Deutsche Welle. Available at: https://www.dw.com/en/japan-whats-behind-okinawans-falling-life-expectancy/a-62088176 (2022, accessed 12 May 2025).

[8] Sjölander P Hassler S Janlert U. Stroke and acute myocardial infarction in the Swedish Sami population: incidence and mortality in relation to income and level of education. Scand J Public Health 2008; 36: 84–91. 18426788 10.1177/1403494807085305

[9] Fujiwara K Osanai T Kobayashi E , et al. Accessibility to tertiary stroke centers in Hokkaido, Japan: use of novel metrics to assess acute stroke care quality. J Stroke Cerebrovasc Dis 2018; 27: 177–184. 28911996 10.1016/j.jstrokecerebrovasdis.2017.08.013

[10] Ishikawa T Mizuguchi H Murayama H , et al. Relationship between accessibility and resources to treat acute ischemic stroke. Hokkaido, Japan: analysis of inequality and coverage using geographic information systems. Health Policy and Technology 2019; 8: 337–342.

[11] Denison HJ Corbin M Douwes J , et al. Ethnic differences in stroke outcomes in Aotearoa New Zealand: a national linkage study. Int J Stroke 2023; 18: 663–671. 36872640 10.1177/17474930231164024 PMC10311930

[12] Thompson SG Barber PA Gommans JH , et al. The impact of ethnicity on stroke care access and patient outcomes: a New Zealand nationwide observational study. Lancet Reg Health West Pac 2022; 20: 100358. 35036976 10.1016/j.lanwpc.2021.100358 PMC8743211

[13] Kilkenny MF Harris DM Ritchie EA Price C Cadilhac DA ; National Stroke Foundation. Hospital management and outcomes of stroke in Indigenous Australians: evidence from the 2009 Acute Care National Stroke Audit. Int J Stroke 2013; 8: 164–171. 22299773 10.1111/j.1747-4949.2011.00717.x

[14] Tiedeman C Suthers B Julien B Hackett A Oakley P. Management of stroke in the Australian Indigenous population: from hospitals to communities. Intern Med J 2019; 49: 962–968. 30907045 10.1111/imj.14303

[15] Souto SDR Anderle P Goulart BNG. Racial inequalities in access to rehabilitation after a stroke: study of the Brazilian population. Cien Saude Colet 2022; 27: 1919–1928. 35544819 10.1590/1413-81232022275.09452021

[16] Yu CY Blaine T Panagos PD Kansagra AP. Demographic disparities in proximity to certified stroke care in the United States. Stroke 2021; 52: 2571–2579. 34107732 10.1161/STROKEAHA.121.034493 PMC8316304

[17] Balabanski AH Dos Santos A Woods JA , et al. Incidence of stroke in indigenous populations of countries with a very high human development index: a systematic review. Neurology 2024; 102: e209138. 10.1212/WNL.0000000000209138 38354325

[18] Heath T Shrishail N Wong KH , et al. Trends in American Indian/Alaskan native self-reported stroke prevalence and associated modifiable risk factors in the United States from 2011-2021. J Stroke Cerebrovasc Dis 2024; 33: 107650. 38460776 10.1016/j.jstrokecerebrovasdis.2024.107650 PMC11253029

[19] Global Plan of Action for the Health of Indigenous Peoples. World Health Organisation. Available at: https://www.who.int/initiatives/global-plan-of-action-for-health-of-indigenous-peoples (2023, accessed 12 May 2025).

[20] International Day of the world’s indigenous peoples 9 and August. United Nations. Available at: https://www.un.org/en/observances/Indigenous-day/background (2024, accessed 12 May 2025)

[21] Cobb E. Japan’s forgotten indigenous people. BBC. Available at: https://www.bbc.com/travel/article/20200519-japans-forgotten-indigenous-people (21 May 2020, accessed 12 May 2025)

[22] Nishida N Hirai M Kawamura S , et al. The history of human populations in the Japanese Archipelago inferred from genome-wide SNP data with a special reference to the Ainu and the Ryukyuan populations. Journal of Human Genetics 2012; 57: 787–795. 23135232 10.1038/jhg.2012.114

[23] Pact AIP and Affairs IWGfI Development AFfHRa ASEAN’s indigenous peoples. Available at: https://www.iwgia.org/images/publications/0511_ASEAN_BRIEFING_PAPER_eb.pdf (2010, accessed 12 May 2025)

[24] Norwegian National Human Rights Institution. A human rights-based approach to Sami statistics in Norway. Available at: https://www.nhri.no/wp-content/uploads/2020/08/StatistikkUrfolk_ENG_web_042021.pdf (2020, accessed 12 May 2025)

[25] Kukutai T Taylor J. Indigenous data sovereignty—toward an agenda. Perth, WA, Australia: Australian University Press, 2016.

[26] Smith LT. Decolonizing methodologies: research and indigenous peoples. London and Dunedin: Zed Books Ltd, 1999.

[27] Brodie T Howard NJ Pearson O , et al. Enhancement of scoping review methodology to reflect Aboriginal and Torres Strait Islander ways of knowing, being and doing. Aust N Z J Public Health 2023; 47: 100096. 37972493 10.1016/j.anzjph.2023.100096

[28] Bussalleu A King N Pizango P , et al. Nuya kankantawa (we are feeling healthy): understandings of health and wellbeing among Shawi of the Peruvian Amazon. Soc Sci Med 2021; 281: 114107. 34153933 10.1016/j.socscimed.2021.114107

[29] Huria T Palmer SC Pitama S , et al. Consolidated criteria for strengthening reporting of health research involving indigenous peoples: the CONSIDER statement. BMC Med Res Methodology 2019; 19: 173. 10.1186/s12874-019-0815-8 PMC6688310 31399058

[30] Sanchez JM Jolly SE Dewland TA , et al. Incident strokes among American Indian individuals with atrial fibrillation. JAMA 2021; 10: e019581. 10.1161/JAHA.120.019581 PMC8174189 33653124

[31] Minority Rights Group. Ainu in Japan. Available at: https://minorityrights.org/communities/ainu/ (2018, accessed 12 May 2025).

[32] Thresia CU Srinivas PN Mohindra KS , et al. The health of indigenous populations in South Asia: a critical review in a critical time. Int J Health Serv 2022; 52: 61–72. 32787539 10.1177/0020731420946588 PMC7611999

[33] Pedersen D Kienzler H Gamarra J. Llaki and ñakary: idioms of distress and suffering among the highland Quechua in the Peruvian Andes. Cult Med Psychiatry 2010; 34: 279–300. 20405314 10.1007/s11013-010-9173-z

[34] Santos AD Mohr K Jude M , et al. Prospective analysis of stroke recognition, stroke risk factors, thrombolysis rates and outcomes in Indigenous Australians from a large rural referral hospital. Intern Med J 2022; 52: 468–473. 33012066 10.1111/imj.15080

[35] Cochrane F Siyambalapitiya S Cornwell P. Assessment and rehabilitation of acquired communication disorders in Aboriginal and Torres Strait Islander adults with stroke or traumatic brain injury: a retrospective chart review. Disability and Rehab 2023; 45: 1154–1164. 10.1080/09638288.2022.2055160 35343342

[36] Samuels I Wang MTM Chong KP . Ethnic differences in access to stroke reperfusion therapy in Northern New Zealand. Neuroepidemiology 2020; 54: 427–432. 32957111 10.1159/000510505

[37] Kelly J Dowling A Hillier S , et al. Perspectives on rehabilitation for Aboriginal people with stroke: a qualitative study. Topics in Stroke Rehab 2022; 29: 295–309. 10.1080/10749357.2021.1911771 34180366

[38] Armstrong E Coffin J Hersh D , et al. “You felt like a prisoner in your own self, trapped”: the experiences of Aboriginal people with acquired communication disorders. Disabil Rehabil 2021; 43: 1903–1916. 31692386 10.1080/09638288.2019.1686073

[39] Cochrane F Singleton-Bray J Canendo W Cornwell P Siyambalapitiya S. “Working together. . . I can’t stress how important it is”: indigenous Health Liaison Officers’ insights into working with speech-language pathologists and Aboriginal and Torres Strait Islander peoples with stroke and TBI. Int J Speech Lang Pathol 2024; 26: 149–161. 37552611 10.1080/17549507.2023.2181225

[40] Quigley R Mann J Robertson J Bonython-Ericson S. Are we there yet? Exploring the journey to quality stroke care for Aboriginal and Torres Strait Islander peoples in rural and remote Queensland. Rural Remote Health 2019; 19: 4850. 31487467 10.22605/RRH4850

[41] Eustace M McGarr K Theys C. Māori aspirations following stroke: a pathway forward for the speech-language therapy field. Aphasiology 2024; 38: 144–167.

[42] Hersh D Armstrong E Panak V Coombes J. Speech-language pathology practices with Indigenous Australians with acquired communication disorders. Int J Speech Lang Pathol 2015; 17: 74–85. 25112423 10.3109/17549507.2014.923510

[43] Legg C Penn P. A stroke of misfortune: cultural intepretations of aphasia in South Africa. Aphasiology 2013; 27: 126–144.

[44] Liao ZY Kean S Haycock-Stuart E. Indigenous lands and health access: the influence of a sense of place on disparities in post-stroke recovery in Taiwan. Health Place 2024; 86: 103210. 38354468 10.1016/j.healthplace.2024.103210

[45] Peake RM Jackson D Lea J Usher K. Meaningful engagement with Aboriginal communities using participatory action research to develop culturally appropriate health resources. J Transcult Nurs 2021; 32: 129–136. 31948353 10.1177/1043659619899999

[46] Armstrong EM Ciccone N Hersh D , et al. Development of the Aboriginal Communication Assessment After Brain Injury (ACAABI): a screening tool for identifying acquired communication disorders in Aboriginal Australians. Int J Speech Lang Pathol 2017; 19: 297–308. 28425776 10.1080/17549507.2017.1290136

[47] Armstrong E McAllister M Hersh D , et al. A screening tool for acquired communication disorders in Aboriginal Australians after brain injury: lessons learned from the pilot phase. Aphasiology 2020; 34: 1388–1412.

[48] Armstrong E Colegate K Papertalk L , et al. Intersectionality and its relevance in the context of aboriginal people with brain injury in Australia. Semin Speech Lang 2024; 45: 56–70. 37992734 10.1055/s-0043-1776755

[49] Drew N McAllister M Coffin J , et al. Healing right way randomized control trial enhancing rehabilitation services for Aboriginal people with brain injury in Western Australia: translation principles and activities. Brain Impairment 2024; 25: IB23109. 10.1071/IB23109 38640359

[50] Katzenellenbogen JM White J Robinson M , et al. Process evaluation of a randomized controlled trial intervention designed to improve rehabilitation services for Aboriginal Australians after brain injury: the Healing Right Way Trial. BMC Health Serv Res 2024; 24: 946. 39164676 10.1186/s12913-024-11390-5 PMC11334317

[51] Armstrong E Coffin J Hersh D , et al. Healing Right Way: study protocol for a stepped wedge cluster randomized controlled trial to enhance rehabilitation services and improve quality of life in Aboriginal Australians after brain injury. BMJ Open 2021; 11: e045898. 10.1136/bmjopen-2020-045898 PMC8479943 34588230

[52] Armstrong E Mcallister M Coffin J , et al. Communication services for First Nations peoples after stroke and traumatic brain injury: alignment of sustainable development goals 3, 16 and 17. Int J Speech Lang Pathol 2023; 25: 147–151. 36412124 10.1080/17549507.2022.2145356

[53] Brewer KM McCann CM Harwood MLN . Working with Maori adults with aphasia: an online professional development course for speech-language therapists. Aphasiology 2020; 34: 1413–1431.

[54] Møller H Baxter R Denton A , et al. Outcomes from a collaborative project developing and evaluating a community rehabilitation worker program for Northwestern Ontario First Nations. Rural Remote Health 2023; 23: 7809. 37429740 10.22605/RRH7809

[55] Morii Y Osanai T Ishikawa T , et al. Cost effectiveness of drive and retrieve system in hokkaido for acute ischemic stroke patient treatment using geographic information system. J Stroke Cerebrovasc Dis 2019; 28: 2292–2301. 31200963 10.1016/j.jstrokecerebrovasdis.2019.05.020

[56] Park SU Cho SY Park JM , et al. Integrative treatment modalities for stoke victims in Korea. Complement Ther Clin Pract 2014; 20: 37–41. 24439643 10.1016/j.ctcp.2013.10.007

[57] Hill ME Bodnar P Fenton R , et al. Teach our children: stroke education for indigenous children, First Nations, Ontario, Canada, 2009–2012. Prev Chronic Dis 2017; 14: E68. 10.5888/pcd14.160506 PMC5716642 28817789

[58] Harwood M Weatherall M Talemaitoga A , et al. Taking charge after stroke: promoting self-directed rehabilitation to improve quality of life—a randomized controlled trial. Clin Rehabil 2012; 26: 493–501. 22087047 10.1177/0269215511426017

[59] Harwood M Weatherall M Talemaitoga A , et al. An assessment of the Hua Oranga outcome instrument and comparison to other outcome measures in an intervention study with Maori and Pacific people following stroke. NZ Med J 2012; 125: 57–67. 23242398

[60] Ranta A Davis A Tyson A , et al. National stroke clot retrieval service improvement programme, 2023. Available at: https://www.tewhatuora.govt.nz/health-services-and-programmes/national-stroke-clot-retrieval-service-improvement-programme (2024, accessed 30 August 2024).

[61] Oser CS Gohdes D Fogle CC , et al. Cooperative strategies to develop effective stroke and heart attack awareness Oser messages in rural American Indian communities, 2009–2010. Preventing Chronic Dis 2013; 10: 120277. 10.5888/pcd10.120277 PMC3666974 23680509

[62] Murad W Ahmad A Gilani SA , et al. Indigenous knowledge and folk use of medicinal plants by the tribal communities of Hazar Nao forest, Malakand district, North Pakistan. J Med Plants Res 2011; 5: 1072–1086. Available at: https://www.scopus.com/inward/record.uri?eid=2-s2.0-79955558319&partnerID=40&md5=5f54223916112fe67ac9a3c005fb89db

[63] Jagtap SD Deokule SS Bhosle SV. Some unique ethnomedicinal uses of plants used by the Korku tribe of Amravati district of Maharashtra, India. J Ethnopharmacology 2006; 107: 463–469. 16713158 10.1016/j.jep.2006.04.002

[64] Walter S Fassbender K Easton D , et al. Stroke care equity in rural and remote areas—novel strategies. Vessel Plus 2021; 5: 27.

[65] Wakuta N Yamamoto S Adachi S Motonaga E. Toward inter-isolated island cooperation for the drip, ship, and retrieve method in the Sakishima islands: a case report. J Neuroendovasc Ther 2020; 14: 263–267. 37502616 10.5797/jnet.cr.2020-0028 PMC10370518

[66] Stroke Foundation of New Zealand. New Zealand clinical guidelines for stroke management 2010. Wellington: Stroke Foundation of New Zealand, 2010, pp. 49–56.

[67] Heart Stroke. Helping to close the gap in Indigenous health. Available at: https://www.heartandstroke.ca/articles/small-stroke-big-wake-up-call (accessed 12 May 2025)

[68] Penn C Armstrong E Brewer K , et al. Decolonizing speech-language pathology practice in acquired neurogenic disorders. Perspectives of the ASHA Special Interest Groups 2017; 2: 91–99.

[69] Ranta A Jones B Harwood M. Stroke among Māori in Aotearoa New Zealand and solutions to address persistent inequities. Frontiers in Stroke 2023; 2: 1248351. 10.3389/fstro.2023.1248351 PMC12802640 41541116

[70] Blacker D Armstrong E. Indigenous stroke care: differences, challenges and a need for change. Intern Med J 2019; 49: 945–947. 31387153 10.1111/imj.14399

[71] Peake RM Jackson D Lea J Usher K. Investigating the processes used to develop and evaluate the effectiveness of health education resources for adult Indigenous people: a literature review. Contemp Nurse 2019; 55: 421–449. 31210593 10.1080/10376178.2019.1633939

[72] United Nations. United Nations declaration on the rights of indigenous peoples. Available at: https://www.un.org/development/desa/indigenouspeoples/wp-content/uploads/sites/19/2018/11/UNDRIP_E_web.pdf (2018, accessed 30 October 2024).

[73] Stavenhagen R . Indigenous peoples and the State in Latin America: an ongoing Debate. In: Sieder R (ed.) Multiculturalism in Latin America: Indigenous Rights, Diversity and Democracy. London: Palgrave Macmillan, 2002, pp. 24–44.

[74] Zhang Y Galloway JM Welty TK , et al. Incidence and risk factors for stroke in American Indians: the Strong Heart Study. Circulation 2008; 118: 1577–1584. 18809797 10.1161/CIRCULATIONAHA.108.772285 PMC2754380

[75] Beans JA Saunkeah B Brian Woodbury R Ketchum TS Spicer PG Hiratsuka VY. Community protections in American Indian and Alaska Native participatory research—a scoping review. Soc Sci (Basel) 2019; 8: 127. 31463160 10.3390/socsci8040127 PMC6713452

[76] Kyllingstad JR. Norwegian physical anthropology and the Idea of a Nordic master race. Current Anthropology 2012; 53: S46–S56.

[77] Cochrane F Singleton-Bray J Canendo W Cornwell P Siyambalapitiya S. “Working together. . . I can’t stress how important it is”: indigenous health liaison officers’ insights into working with speech-language pathologists and Aboriginal and Torres Strait Islander peoples with stroke and TBI. Int J Speech Lang Pathol 2024; 26: 149–161. 37552611 10.1080/17549507.2023.2181225

[78] Liao Z-Y Haycock-Stuart E Kean S. Biographical continuation: recovery of stroke survivors and their family caregivers in Taiwan. Primary Health Care Res and Dev 2024; 25: e2. 10.1017/S1463423623000610 PMC10790715 38179717

[79] Hersh D Armstrong E Bourke N. A narrative analysis of a speech pathologist’s work with Indigenous Australians with acquired communication disorders. Disabil Rehabil 2015; 37: 33–40. 24564327 10.3109/09638288.2014.890675

[80] Shankar J Dian J Trivedi R Linton J. Clinical outcomes of ischemic stroke in indigenous populations—A systematic review and metanalysis. Int J Stroke 2023; 18: 95.35120419

[81] World Intellectual Property Organisation. Genetic resources, traditional knoweldge and traditional cultural expressions. Available at: https://www.wipo.int/tk/en/ (2024, accessed 12 May 2025).

[82] Dos Santos A Cheong E Balabanski AH , et al. First stroke incidence, causes, treatments, and outcomes for Aboriginal Peoples in South Australia and the Northern Territory: a pilot prospective study. Med J Aust 2024; 221: 39–46. 38946653 10.5694/mja2.52356

[83] Hart M Dos Santos A Leclair L , et al. Improving indigenous stroke outcomes by shifting our focus from health to cultural literacy. Curr Neurol Neurosci Rep 2024; 25: 3. 39565456 10.1007/s11910-024-01395-2

[84] Gordon C Bell R Ranta A. Impact of the national public “FAST” campaigns. NZ Med J 2019; 132: 48–56. Available at: https://www.ncbi.nlm.nih.gov/pubmed/31830016 31830016

[85] Smith GH. The development of kaupapa Maori: theory and praxis. University of Auckland. Available at: https://researchspace.auckland.ac.nz/handle/2292/623 (1997, accessed 12 May 2025).

[86] Boland P Jones B Stanley J , et al. Using a maori model of health to analyse the use of equipment by new zealand maori post-stroke. NZ J Occ Ther 2020; 67: 19–26.

[87] Bessarab D Ng’andu B. Yarning about yarning as a legitimate method in indigenous research. Int J Crit Indigenous Stud 2010; 3: 37–50.

[88] Curtis E Wikaire E Stokes K , et al. Addressing indigenous health workforce inequities: a literature review exploring “best” practice for recruitment into tertiary health programmes. Int J Equity in Health 2012; 11: 13. 22416784 10.1186/1475-9276-11-13 PMC3402985

[89] Owusu-Ansah S Tripp R Weisberg SN Mercer MP Whitten-Chung K ; NAEMSP Diversity Equity, Inclusion Committee. Essential principles to create an equitable, inclusive, and diverse EMS workforce and work environment: a position statement and resource document. Prehosp Emerg Care 2023; 27: 552–556. 36867425 10.1080/10903127.2023.2187103

[90] Taylor EV Lyford M Parsons L Mason T Sabesan S Thompson SC. “We’re very much part of the team here”: a culture of respect for Indigenous health workforce transforms Indigenous health care. PLoS ONE 2020; 15: e0239207. 10.1371/journal.pone.0239207 PMC7508383 32960933

[91] Petrov AN Dorough DS Tiwari S , et al. Indigenous health-care sovereignty defines resilience to the COVID-19 pandemic. Lancet 2023; 401: 1478–1480. 37084754 10.1016/S0140-6736(23)00684-0 PMC10112862

